# *Trypanosoma cruzi* Transmission Among Captive Nonhuman Primates, Wildlife, and Vectors

**DOI:** 10.1007/s10393-018-1318-5

**Published:** 2018-03-01

**Authors:** Carolyn L. Hodo, Gregory K. Wilkerson, Elise C. Birkner, Stanton B. Gray, Sarah A. Hamer

**Affiliations:** 1 Veterinary Integrative Biosciences Department, Texas A&M University College of Veterinary Medicine and Biomedical Research, College Station, TX 77843-4458 USA; 2 MD Anderson Cancer Center, Michale E. Keeling Center for Comparative Medicine and Research, Bastrop, TX USA

**Keywords:** *Trypanosoma cruzi*, chagas disease, *Macaca mulatta*, triatomine, Texas, wildlife

## Abstract

Natural infection of captive nonhuman primates (NHPs) with *Trypanosoma cruzi* (agent of Chagas disease) is an increasingly recognized problem in facilities across the southern USA, with negative consequences for NHP health and biomedical research. We explored a central Texas NHP facility as a nidus of transmission by characterizing parasite discrete typing units (DTU) in seropositive rhesus macaques (*Macaca mulatta*), identifying the wildlife reservoirs, and characterizing vector infection. In seropositive NHPs, we documented low and intermittent concentrations of circulating *T. cruzi* DNA, with two DTUs in equal proportions, TcI and TcIV. In contrast, consistently high concentrations of *T. cruzi* DNA were found in wild mesomammals at the facility, yet rodents were PCR-negative. Strong wildlife host associations were found in which raccoons (*Procyon lotor*) harbored TcIV and opossums (*Didelphis virginiana*) harbored TcI, while skunks (*Mephitis mephitis*) were infected with both DTUs. Active and passive vector surveillance yielded three species of triatomines from the facility and in proximity to the NHP enclosures, with 17% *T. cruzi* infection prevalence. Interventions to protect NHP and human health must focus on interrupting spillover from the robust sylvatic transmission in the surrounding environment.

## Introduction

*Trypanosoma cruzi*, the zoonotic vector-borne agent of Chagas disease, is widespread throughout the Americas as far north as the southern USA, infecting over 200 species of mammals. The complexity of sylvatic transmission cycles, which involve multiple genetic strains of the parasite maintained by a diverse community of wildlife hosts, presents one of the major challenges to Chagas disease control and prevention. *T. cruzi* is divided into 7 genetic strain types or discrete typing units (DTUs): TcI–TcVI and TcBat, which are reportedly associated with differing clinical manifestations, reservoir host species, and geographical locations (Barr et al. 1991a, b; Ramírez et al. 2010; Duz et al. 2014; Jansen et al. 2015). In the USA, TcI and TcIV are the most commonly reported DTUs (Bern et al. 2011; Roellig et al. 2013), and diverse mammalian wildlife species serve as reservoirs (Bern et al. 2011; Zeledón et al. 2012; Hodo and Hamer 2017).

*Trypanosoma cruzi* is transmitted by triatomine bugs, which acquire infection by blood feeding on an infected mammal. The infective stage of the parasite is passed in the bug’s feces which contaminates the bite wound or nearby mucous membranes in subsequent hosts. Additionally, oral transmission through the ingestion of infected bugs is important for animals (Barr 2009; Dorn et al. 2012; Rocha et al. 2013; Desquesnes 2017). Transmission can also occur congenitally and through blood transfusion or organ transplant.

Some free-ranging neotropical nonhuman primate (NHP) species are important sylvatic hosts of *T. cruzi* (Lisboa et al. 2015; Jansen et al. 2017), and natural infection is described in captive New and Old World NHPs in areas where vectors are found (Williams et al. 2009; Bommineni et al. 2009; Dorn et al. 2012; Minuzzi-Souza et al. 2016). As in humans and dogs, infection in NHPs is characterized by acute, indeterminate, and chronic stages with a subset of infected animals developing a lethal cardiomyopathy or, less commonly, gastrointestinal issues (Bonecini-Almeida et al. 1990; Carvalho et al. 2003; Monteiro et al. 2006). In the USA, natural *T. cruzi* infection of outdoor-housed NHPs has been reported since the 1970s (Cicmanec et al. 1974; Kasa et al. 1977). Published surveys report infection prevalence ranging from 2 to 10% in NHP facilities in the southern USA (Kasa et al. 1977; Dorn et al. 2012; Pisharath et al. 2013), and both DTUs TcI and TcIV have been documented (Roellig et al. 2013). Because animals are often transported across the country from the South, *T. cruzi* infection is a concern in NHPs housed in nonendemic areas as well (Dickerson et al. 2014).

Incidental infection of captive NHPs presents significant problems for biomedical research. Undetected infection with *T. cruzi* or other agents compromises the integrity of the NHP research model by introducing unpredictable variability into studies at great cost to the biomedical industry. Seropositive NHPs may be removed from the pool of animals used in research and breeding, due not only to the potential confounding effects of infection, but also to concerns over the potential to spread the infection to other NHPs. However, the actual risk posed by seropositive NHPs to others in the colony has yet to be characterized. Additionally, little is known about the ecological interface between *T. cruzi* transmission among captive NHPs and wildlife in the surrounding sylvatic environment.

In the absence of effective vaccines and drugs against chronic *T. cruzi* infections, interventions must be aimed at preventing infection by interrupting vector-mediated spillover from the sylvatic cycle. Additionally, to develop informed guidelines for the management of infected NHPs, it is necessary to quantify the infectious potential of seropositive animals to determine whether they may serve as reservoirs. Thus, the objectives of this study were to characterize the transmission cycles of *T. cruzi* at a NHP facility with approximately 4% seroprevalence in rhesus macaques by (1) determining the presence and DTUs of *T. cruzi* DNA circulating in the blood of seropositive macaques; (2) identifying the local wildlife reservoirs that are most important in infecting vectors that may contact NHPs; and (3) documenting the presence and infection status of triatomine vectors.

## Materials and Methods

### Facility

**Figure 1:**
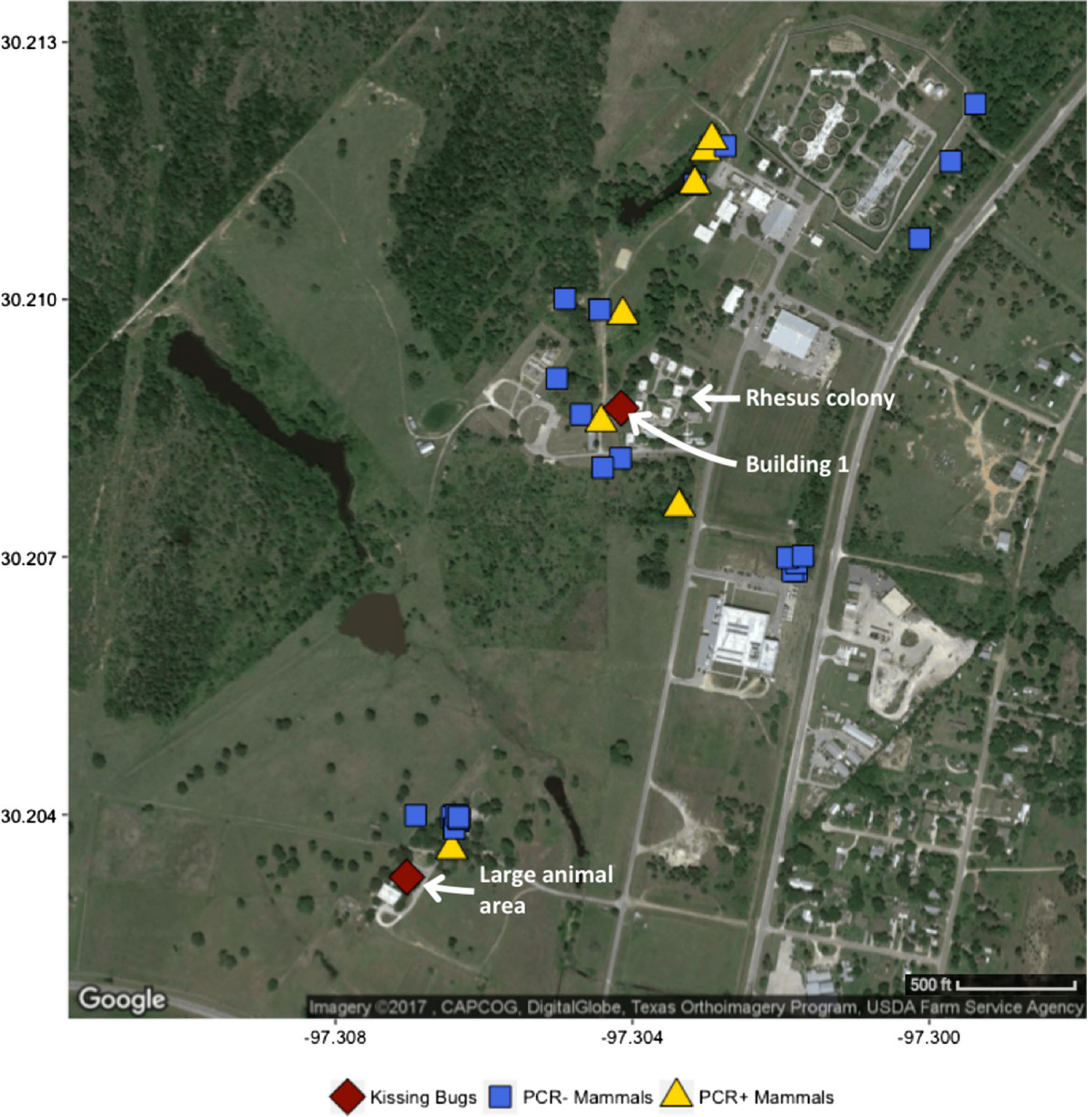
Satellite image of KCCMR facility indicating the locations where *T. cruzi* PCR-positive and PCR-negative mammals were trapped and where kissing bugs were found. The location of the rhesus colony housing and the two sites where kissing bugs were collected are labeled with white text and arrows. Created in R using ggplot2 and ggmap packages (Kahle and Wickham 2013). Google map image from: http://maps.googleapis.com/maps/api/staticmap?center=30.20756,-97.30475&zoom=16&size=640x640&scale=2&maptype=satellite&language=en-EN&sensor=false.

The MD Anderson Cancer Center Michale E. Keeling Center for Comparative Medicine and Research (KCCMR) is located on 381 mostly wooded acres in Bastrop county in central Texas (Fig. 1). In addition to several other primate species, the facility maintains an Indian-origin rhesus macaque (*Macaca mulatta*) breeding colony housed in open-air enclosures. The KCCMR is fully accredited by the Association for Assessment and Accreditation of Laboratory Animal Care International and animals are housed in compliance with the recommendations in the *Guide for the Care and Use of Laboratory Animals* (National Research Council 2011).

### Primates

The KCCMR rhesus macaque breeding colony is comprised of approximately 980 animals and has been a closed colony with no outside additions since 1983. The colony has been documented through serological means to be specific pathogen free (SPF) for cercopithecine herpesvirus 1, simian immunodeficiency virus, systemic T-lymphotropic virus, and simian retroviruses 1, 2, and 5 since 1991. Surveillance for *T. cruzi* in the macaque colony was first performed in 2013 and has been conducted yearly since 2015, with resulting colony-wide seroprevalence of approximately 4%. Preliminary review of records has not identified any familial associations of seropositivity, and none of the seropositive animals in this study are the offspring of known infected mothers. All macaques used in this study were determined to be seropositive for *T. cruzi* for at least two consecutive years using a suspension microarray from a commercial laboratory (Macaque Chagas Multiplexed Fluorometric ImmunoAssay, Charles River Laboratories, Wilmington, MA), run in conjunction with an ELISA at the same commercial laboratory.

From July 2016 to January 2017, we collected up to 2 ml of whole blood from 41 seropositive animals and performed a series of PCRs to determine the presence and DTU of *T. cruzi* DNA in the blood. Each animal that was not positive on both the screening and confirmatory qPCRs was resampled at the next opportunity 1–2 months later in an iterative process, with each seropositive animal being sampled from 1 to 4 times. In some cases, animals were resampled even when DTU was determined from a previous sample to evaluate consistency in blood infection status over time. All NHP blood collections were approved through the University of Texas, MD Anderson Cancer Center Institutional Animal Care and Use Committee.

DNA was extracted from 1 ml of previously frozen EDTA-treated whole blood using a commercial spin-column extraction kit (E.Z.N.A. Tissue DNA kit, Omega Bio-Tek, Norcross, GA) according to the manufacturer’s tissue extraction protocol but with a final elution volume of 50 µl and following instructions for scaling up to larger volumes of starting material. Extracted DNA was first subjected to a screening, real-time quantitative PCR (qPCR) for the specific detection of *T. cruzi* using the Cruzi 1/2 primers and a 6-carboxyfluorescein(FAM)-labeled probe, Cruzi 3, as previously described (Piron et al. 2007; Ramírez et al. 2015), but with an initial denaturation time of 3 min. This assay has been shown to be a best-performing method in an international PCR study (Schijman et al. 2011) and is sensitive and specific for all DTUs of *T. cruzi* (Ramírez et al. 2015). Based on internal laboratory validations, the cutoff for suspect positive samples was determined to be a quantification cycle threshold (Ct) value ≤ 34. DNA-negative (water) controls and a positive control of DNA extracted from pure culture of Sylvio X10 CL4 (ATCC 50800, American Type Culture Collection, Manassas, VA; DTU TcI) were included in all reactions. A 7-point standard dilution series (10^0^–10^6^ dilutions) of extracted DNA was made from a known concentration of parasite determined by hemocytometer and used in the qPCR to allow for estimation of parasite concentration in samples. Next, all samples that screened positive, as well as some that were negative, were subjected to a multiplex probe-based qPCR for determination of DTU (Cura et al. 2015). This assay is highly sensitive for all known DTUs with an analytical sensitivity of 1 fg to 1 pg of DNA/reaction tube (Cura et al. 2015). Negative controls (water) and positive controls of DNA extracted from *T. cruzi* strain Sylvio X10 CL4 (DTU TcI) and *T. cruzi*-infected *Triatoma sanguisuga* from Texas (DTU TcIV) were included in all reactions, and positive control of DNA extracted from *T. cruzi* Y-strain (ATCC 50832, American Type Culture Collection, Manassas, VA; DTU TcII) was added for reactions run later in the study. Samples that were suspect positive in the screening qPCR yet negative on the SL-IR qPCR under standard conditions were rerun using two additional treatments: (1) 1:10 dilution of the DNA template, and (2) 2 times the volume of DNA template to afford additional opportunity to ascertain the DTU.

For statistical analysis, NHP samples were considered PCR positive when they were positive on both qPCRs. Bivariable analysis using Chi-squared or Fisher’s exact tests was used to assess relationships between NHP age (≤ 16 or > 17 years; *n* = 22 individuals per group) and sex with blood PCR status and DTU. All risk factors with *P* value ≤ 0.25 in bivariable analysis were further investigated with logistic regression using generalized linear models. Values of *P* ≤ 0.05 were considered significant. Odds ratios and 95% confidence intervals were calculated for the risk factors. An exact binomial test was used to compare the proportions of NHPs infected with different DTUs, and a Welch’s 2-sample *t* test was used to compare Ct values for macaques and wildlife. All statistical analyses were performed in R (R Core Team 2014).

### Wildlife

We visited the facility for mammal trapping 8 times from July to September 2016 at 1–2-week intervals. Small mammals were trapped using Sherman live traps (H.B. Sherman Traps, Tallahassee, FL) spaced approximately 10-m apart and baited with sunflower seeds. During each of 8 visits, we set 60–160 traps in 3–5 different areas for one night. Medium mammals were trapped using 13–14 Tomahawk live traps (Tomahawk Live Trap, Hazelhurst, WI) baited with a combination of canned cat food, tuna, sardines, peanut butter, bacon grease, and marshmallows. Trap locations were chosen based on the appearance of suitable habitat and previous trapping success (Fig. 1).

The location, species, sex, and weight of all captured mammals were recorded. Small mammals were euthanized via an inhaled overdose of isoflurane anesthetic agent (IsoFlo, Zoetis, Parsippany, NJ), exsanguination was performed via intracardiac puncture, and the heart was collected. In the case of pregnant rodents, fetuses were euthanized individually via intracoelomic injection of potassium chloride (KCl). Medium-sized mammals were weighed in the trap and were anesthetized via an intramuscular injection of tiletamine hydrochloride and zolazepam hydrochloride (Telazol, Zoetis, Parsippany, NJ) at 10 mg/kg for raccoons (*Procyon lotor*) and skunks (*Mephitis mephitis*) (Kreeger and Arnemo 2012), and 30 mg/kg for opossums (*Didelphis virginiana*) (Stoskopf et al. 1999). Anesthetized animals were euthanized via intracardiac injection of 50 mg/kg KCl. Blood (1–3 ml) was collected via intracardiac puncture, and the heart was collected. All blood samples were stored in microcentrifuge or vacutainer tubes with no additives. In addition, we received rodents found dead by the facility’s pest control personnel from which hearts and blood clot were collected. Wildlife research was permitted by Texas Parks and Wildlife Department and approved by animal use committees at Texas A&M University and MD Anderson Cancer Center.

In the laboratory, the serum was separated from the clot. Hearts were examined for gross abnormalities and dissected to allow visualization of all four chambers. Samples were collected for PCR from left and right ventricles and atria. Heart tissue was minced and frozen at − 20°C until extraction. We extracted DNA from 500 µl of blood clot from wildlife species and approximately 0.5 cm^3^ of heart using a commercial extraction kit (E.Z.N.A. Tissue DNA kit, Omega Bio-Tek, Norcross, GA) following the manufacturer’s protocol for tissue extraction with modifications and controls as detailed above. PCRs for *T. cruzi* detection and DTU determination were performed as described above.

### Triatomine Vectors

Active nighttime kissing-bug surveillance was performed during the same eight visits using active searches and stationary white cloth sheets with UV lights and occasionally dry ice. Each night, bug surveillance was conducted by a three-person team for 3–3.5 h beginning around 9:00 pm. Three-to-four vector trapping stations were set up in areas between sylvatic habitat and NHP housing. Each trapping station and the immediate vicinity were actively checked for bugs 3–4 times each hour. Between checks, team members patrolled the facility with flashlights to actively search walls and sidewalks for bugs. Additionally, passive vector surveillance was conducted by facility personnel, who submitted bugs encountered during the course of normal duties over the summer months of 2015 and 2016.

Bugs were identified to species (Lent and Wygodzinsky 1979), measured, sexed, externally decontaminated with 10% bleach, rinsed in distilled water, and dissected to isolate the hindguts. The hindguts were subjected to DNA extraction and PCRs for *T. cruzi* detection and characterization as described above.

## Results

### Primates

**Figure 2:**
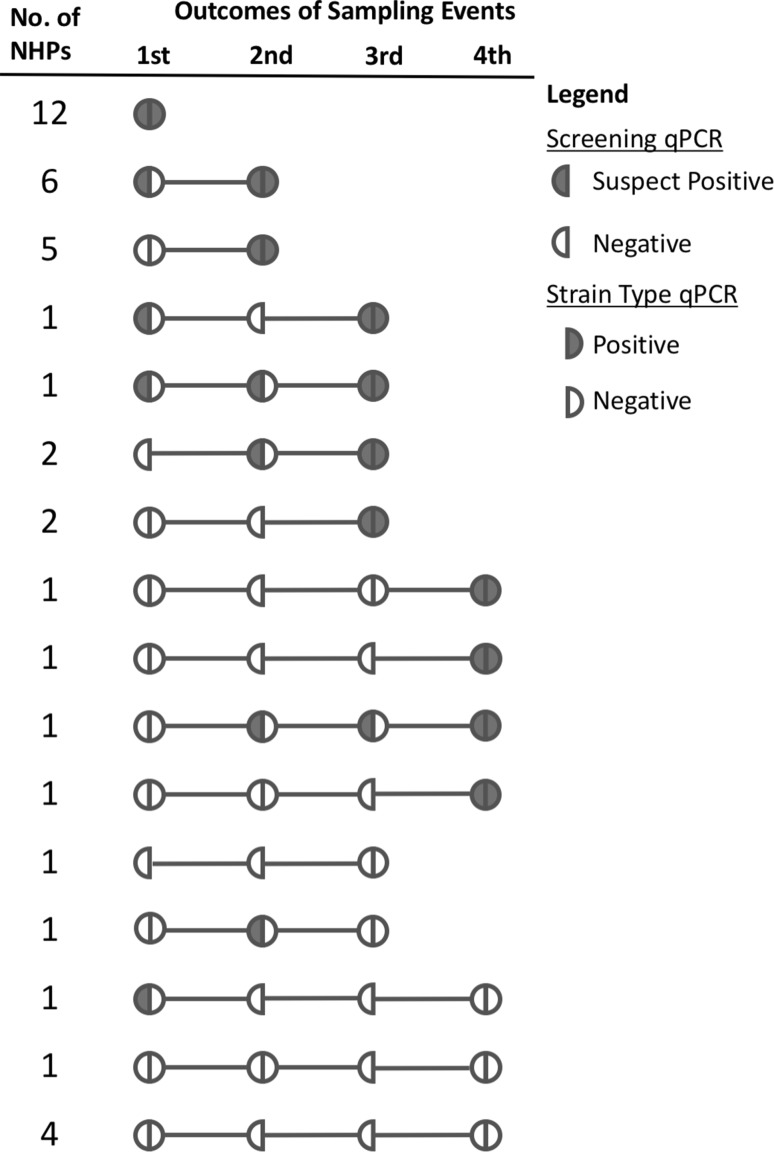
Graphical demonstration of sampling efforts and qPCR results for NHP blood samples. The number of NHPs with each specific sampling and results profile is in the left-hand column. Semicircles depict outcomes of qPCR assays as described in the legend.

**Figure 3:**
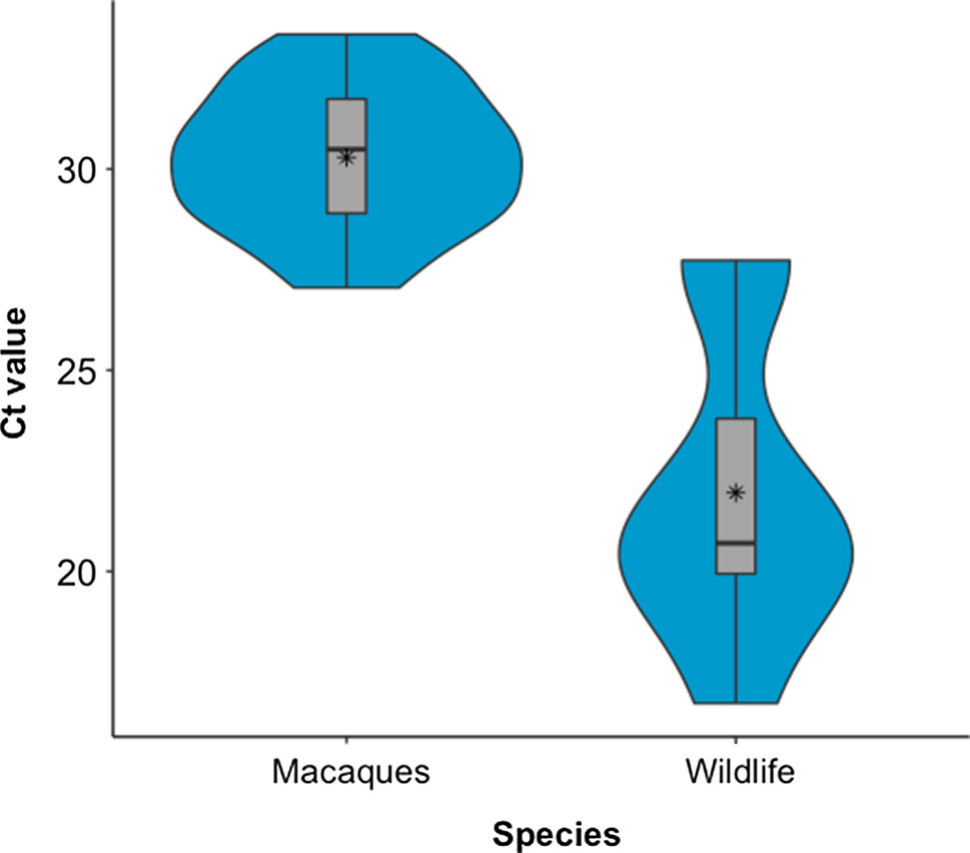
Violin and box plots demonstrating higher Ct values in blood of macaques than in blood of wildlife, representing lower concentrations of circulating parasite DNA in macaques. Mean Ct value (*) for wildlife (21.96) is equivalent to approximately 1500 parasites/ml while mean Ct value (*) for macaques (30.29) is equivalent to approximately 15 parasites/ml.

All known seropositive macaques housed at the facility at the onset of the study (*n* = 41) were sampled, including 31 females (73%) and 10 males (27%) with age range 4–23 years (Table 1). The sex distribution of these seropositive macaques roughly equals the sex distribution within the entire colony, which has an approximate female-to-male ratio of 4:1. Because our goal was to ascertain circulating parasite DTU in seropositive individuals, which often required repeated blood draws to detect parasite DNA, we sampled 14 animals once, 10 animals twice, 8 animals three times, and 13 animals four times (Fig. 2). At the end of the study, 33/41 (80%) seropositive macaques had at least one PCR-positive blood sample for which the DTU was determined (Table 1). Five of these animals were re-sampled one additional time after determination of DTU (from 3 to 9 weeks later), and of those, only one was still positive on both qPCRs, two were suspect positive on the screening qPCR but negative on the strain-typing qPCR, and two were negative on the screening qPCR. Of the eight seropositive macaques for which a DTU was not determined, two had a suspect positive result on the screening qPCR but were negative on multiple attempts of the typing assay and six were PCR-negative across three-to-four different blood samples (Fig. 2). In animals confirmed as PCR positive with DTU determined, Ct values on the screening qPCR ranged from 27 to 33.35, equivalent to 150 to 1.5 parasite equivalents/ml (Fig. 3) as estimated from the standard curve.

**Table 1:** Demographic Data and PCR Positivity^a^ of 41 Seropositive Rhesus Macaques.

	*n*	PCR positive^a^ (%)
Overall	41	33 (80%)
Age (years)		
19–23	15	13 (87%)
15–18	10	10 (100%)
10–12	11	6 (55%)
4–8	5	4 (80%)
Sex		
Female	31	27 (87%)
Male	10	6 (60%)
Strain type (DTU)		
TcI	18	
TcIV	13	
TcI + TcIV	2	

^a^PCR-positive status defined as positive on both the screening and strain-typing qPCR.

In bivariable analysis to determine significant predictors of NHP PCR-positive status among seropositive individuals, age group and sex had *P* values of 0.053 and 0.08, respectively, and were included in the logistic regression model. As estimated by logistic regression, neither age group (*P* = 0.13) nor sex (*P* = 0.057) were significantly associated with PCR-positive status.

Of the NHPs, 13 females and five males were infected with TcI, 13 females with TcIV, and one male and one female were co-infected with both TcI and TcIV. There was no difference in infection of NHPs by TcI (*n* = 18) versus TcIV (*n* = 14; *P* = 0.45). A significant association between sex and DTU was detected (*P* = 0.045), in which no male NHPs were infected with TcIV alone.

### Wildlife

Over eight visits to the facility (780 total trap nights), we captured 38 mammals of five species in multiple locations across the facility grounds, all less than 0.5 mile from the rhesus colony (Fig. 1). We also received eight roof rats (*Rattus rattus*) and one white-footed mouse (*Peromyscus leucopus*) from pest control personnel (Table 2). Overall, 8 of 10 mesomammals were PCR-positive for *T. cruzi* in blood, including 6 which also had infected heart tissue, whereas *T. cruzi* DNA was not detected in hearts or blood of any of four rodent species (Table 2). All 8 infected mesomammals had *T. cruzi* DNA circulating in the blood, with Ct values ranging from 16 to 27 (Fig. 3), equal to approximately 150,000 to 150 parasite equivalents/ml. The mean Ct value for wildlife (21.96), representing approximately 1500 parasites/ml, was significantly lower than the mean Ct value for macaques (30.29), representing approximately 15 parasites/ml (*P* < 0.001; Fig. 3). Both raccoons were infected with DTU TcIV, and the four *T. cruzi*-positive opossums were infected with TcI (Table 2). Of the two positive skunks, one was infected with TcI and the other with TcIV (Table 2).

**Table 2:** Mammals Collected from Primate Facility Grounds Summer 2016, with Results of *T. cruzi* qPCR on DNA Extracted from Heart Tissue and Blood Clot.

Species	Common name	Overall no. *T. cruzi* qPCR positive/no. tested	No. with positive heart	No. with positive blood	*T. cruzi* Strain type (DTU)
*Didelphis virginiana*	Virginia opossum	4/5	3/5	4/5	TcI
*Procyon lotor*	Raccoon	2/5	1/2	2/2	TcIV
*Mephitis mephitis*	Striped skunk	2/3	2/3	2/3	TcI, TcIV
*Sigmodon hispidus*	Cotton rat	0/27	0/27	0/27	–
*Neotoma floridana*	Woodrat	0/1	0/1	0/1	–
*Rattus rattus*	Roof rat	0/8	0/8		–
*Peromyscus* sp.	Mouse	0/1	0/1		

### Triatomine Vectors

Six bugs were collected from the facility from September 2015 to August 2016. Two were collected across 25 h of active vector surveillance over 8 nights from July to September 2016 and four were collected during passive surveillance by facility staff in 2015 and 2016 (Table 3). Five of the six bugs were collected from a single building which houses shower/locker rooms (Building 1) located just on the edge of the rhesus colony bordering sylvatic habitat with thick underbrush (Fig. 1). This building had interior and exterior lights that often remained on at night, and an exterior door that occasionally did not close tightly. *Triatoma sanguisuga* (*n* = 4) was the species most often collected (Table 3) and we also collected 1 each of *T. gerstaeckeri* and *T. lecticularia*. A single *T. sanguisuga* was PCR-positive for *T. cruzi* DTU TcI (17% prevalence).

**Table 3:** Kissing Bugs Collected from the Primate Facility Grounds 2015–2016.

Species	Sex	Month	Location	*T. cruzi* status
*Triatoma sanguisuga*	M	September 2015	Building 1 shower room	Neg
*Triatoma gerstaeckeri*	F	September 2015	Building 1 shower room	Neg
*Triatoma sanguisuga*	F	September 2015	Large animal	Neg
*Triatoma lecticularia*	M	June 2016	Building 1 shower room	Neg
*Triatoma sanguisuga*	M	July 2016	Outside building 1	Pos, TcI
*Triatoma sanguisuga*	F	August 2016	Building 1 entry	Neg

## Discussion

*Trypanosoma cruzi* infection in NHPs in biomedical research facilities has important implications for the health of the animals and for their use as research models. Our findings illustrate a robust transmission cycle of *T. cruzi* involving NHPs, multiple local wildlife species, and triatomine vectors on the campus of a nonhuman primate facility in central Texas. Importantly, we document high concentrations of *T. cruzi* DNA in the blood of infected mesomammals in close proximity to NHP enclosures (Fig. 1). While PCR does not demonstrate the presence of whole, viable parasites, PCR-positive blood samples suggest that the animal could be parasitemic and thus serve as a source of infection to blood-feeding kissing bug vectors. We found that the infected medium-sized wild mammals had concentrations of *T. cruzi* DNA circulating in their blood that were orders of magnitude higher than the NHPs (Fig. 3). Further, whereas the wildlife blood infections were detected with a single blood draw, NHP blood infections were detected only intermittently in some individuals (Fig. 2). Our findings suggest that mesomammals such as raccoons, opossums, and skunks in close proximity to NHP facilities likely play important roles as local reservoirs of *T. cruzi*, while NHPs themselves may be less likely to be infectious to vectors.

Differences have been reported in the relative sensitivity of detection of *T. cruzi* DNA in different blood components (Fitzwater et al. 2008; Schijman et al. 2011; Qvarnstrom et al. 2012). While we extracted DNA from different components of blood (1 ml whole blood from NHPs and 0.5 ml blood clot in wildlife), differences in the detected load of parasite DNA spanned orders of magnitude (e.g., mean of 1500 vs. 15 parasites/mL in wildlife vs. NHPs, respectively); such large differences are unlikely to be explained by extraction method alone.

We determined the infecting DTU for 80% of seropositive NHPs. Previous reports of DTU in NHPs in the USA are limited, with 2 rhesus macaques in Texas harboring TcI, and free-ranging lemurs in Georgia harboring TcIV (Roellig et al. 2013). TcI was detected in captive NHPs in a zoological park in Brazil (Minuzzi-Souza et al. 2016). We document both DTUs TcI and TcIV in this population of rhesus macaques, with two individuals being co-infected with both DTUs simultaneously. More work is needed to correlate DTU with clinical outcome in NHPs, which could have important implications for management of infected animals, as well as for their use as animal models of *T. cruzi* infection in humans. In the USA, only TcI and isolates from the TcII/V/VI complex have been detected in autochthonous human cases. TcIV has not been documented in humans in the USA (Roellig et al. 2008; Garcia et al. 2017), but has been implicated as the cause of disseminated Chagas disease in a dog in Texas (Curtis-Robles et al. 2018a).

Recognition of the associations between parasite DTU and reservoir host species may have important implications for public or veterinary health (Bern et al. 2011; Jansen et al. 2017; Hodo and Hamer 2017). We found that NHPs were infected almost equally with both TcI and TcIV, whereas the small numbers of raccoons and opossums exclusively harbored TcIV and TcI, respectively—host associations that have been previously shown (Bern et al. 2011; Roellig et al. 2013). Both DTUs have been reported in skunks (Roellig et al. 2008; Charles et al. 2012), and this was consistent with our findings. Accordingly, all these local wildlife species may be important as reservoirs for the strains that spill over from the sylvatic cycle to infect NHPs.

While infection was common in mesomammals, we did not detect *T. cruzi* in the blood or heart of any of the rodent species we tested, even though these animals were captured in the same location and during the same time period as the mesomammals. This is consistent with our findings at another NHP facility in Texas, in which *T. cruzi* was not detected in any of 156 *R. rattus* (Hodo et al. 2017). *T. cruzi* and triatomine bugs have long been associated with woodrats (*Neotoma* spp.) in the USA (Packchanian 1942; Charles et al. 2012), and several studies have reported *T. cruzi* infection in other species of rodents (Burkholder et al. 1980; Navin et al. 1985; Charles et al. 2012; Herrera et al. 2015; Aleman et al. 2017). *T. cruzi* transmission cycles are characterized by regional heterogeneity, and the important reservoirs likely differ across geographical areas (Hodo and Hamer 2017). Because the mammal traps were not placed randomly or systematically along transects, the species and numbers of mammals captured are not necessarily reflective of the entire population structure of the wildlife community. Nevertheless, the lack of detection of parasite DNA in the hearts or blood of the rodents tested, in the face of active infection in other species, suggests that rodents are likely not an important part of the reservoir community at this facility.

We documented the ongoing presence of triatomine vectors at this facility over two summers, though the number of triatomines we collected was low. Triatomines are nocturnal, elusive, and notoriously difficult to collect using standard entomological methods (Kjos et al. 2013; Curtis-Robles et al. 2015). Interestingly, most of the bugs were collected from a single building (Building 1) housing shower rooms on the border between moderately dense sylvatic habitat and the rhesus colony (Fig. 1; Table 3); several NHP enclosures surround this building. While no triatomines were recovered from the enclosures, it is probable that NHPs readily consume insects that enter their environment which would allow for infections yet preclude discovery of the insects by staff members. The relatively low infection prevalence among the bugs we collected (1/6, 17%) compared with other prevalence estimates in Texas (50–63%) (Kjos et al. 2009; Curtis-Robles et al. 2015) is likely an artifact of the small sample size. Of 22 bugs tested from elsewhere in the same county, 15 (68.2%) were *T. cruzi* positive (Curtis-Robles et al. 2018b).

In conclusion, we document active *T. cruzi* transmission at a NHP biomedical research facility, involving macaques, wildlife, and triatomine vectors. Our findings add important components needed to better understand the transmission cycles of *T. cruzi* in the southern USA. Interventions to block transmission of vector-borne diseases such as *T. cruzi* to NHPs should be aimed at interrupting spillover from the sylvatic cycles by limiting contact between NHPs and vectors and potentially by managing surrounding habitat to increase the distance between the sylvatic cycle and NHPs.

Broader implications of our findings highlight the unique ecological and epidemiological situation presented by the outdoor housing of nonnative NHP species with exposure to local vectors. While our studies focused on *T. cruzi*, outdoor-housed NHPs in biomedical research facilities across the southern USA are within endemic transmission ranges of other vector-borne and infectious diseases for which regular NHP testing and surveillance may not occur. Whether or not captive outdoor-housed NHPs could significantly impact the epidemiology of these pathogens is not currently known and warrants further investigation. Our recommendation is that serosurveillance for *T. cruzi* and other vector-borne diseases be incorporated into yearly screening for outdoor-housed NHPs.
